# Diagnosis and treatment of congenital anterior urethral Valves: a retrospective analysis of 8 cases

**DOI:** 10.3389/fsurg.2026.1886182

**Published:** 2026-07-06

**Authors:** Linling Gui, Hui Ma, Tao Zhu, Runwu Sun, Yuan Cai, Ting Gao, Haitao Chen, Xufei Duan

**Affiliations:** 1 Department of Neonatal Surgery, Wuhan Children’s Hospital, Tongji Medical College, Huazhong University of Science &Technology, Wuhan, China; 2 Department of Urology, Wuhan Children’s Hospital, Tongji Medical College, Huazhong University of Science &Technology, Wuhan, China

**Keywords:** anterior urethral valves, outcomes, pediatrics, renal impairment, urethral diverticula

## Abstract

**Background:**

This study aims to elucidate the clinical characteristics, management, and prognosis of anterior urethral valves (AUV) in children.

**Methods:**

A retrospective analysis was performed on 8 pediatric AUV patients (2015–2025), examining their clinical presentation, imaging and renal function data, surgical management, and reoperation rates. Prognosis was evaluated during follow-up.

**Results:**

Among the eight patients, four were diagnosed with AUV (one including posterior urethral valves (PUV)), and four had AUV combined with diverticula (one also associated with PUV). All diagnoses were confirmed by retrograde cystography and/or cystoscopy. Associated findings included bladder trabeculation in four cases and vesicoureteral reflux in four. Three patients had renal impairment. Treatment included cystoscopic electrocoagulation of the urethral valves, or a combination of valve resection, diverticulectomy, and urethral reconstruction. Postoperatively, five patients recovered completely, while one experienced occasional urinary tract infections (UTI). Two patients required reoperation; they continue to exhibit vesicoureteral reflux and renal impairment, and remain under close observation.

**Conclusion:**

AUV can be definitively diagnosed via cystoscopy or voiding cystourethrogram (VCUG). Surgical intervention, including endoscopic valve ablation or open urethroplasty, yields favorable outcomes. However, the prognosis for patients with pre-existing renal impairment remains poor. Consequently, close monitoring and long-term follow-up are imperative.

## 1 Introduction

Anterior urethral valves (AUV) are a rare cause of lower urinary tract obstruction in male children (1, 2). Approximately one-third of cases are associated with a urethral diverticulum, pathologically characterized by an outward protrusion of the anterior urethra through the corpus spongiosum. Approximately 40% of AUVs are located in the bulbar urethra, 30% at the penoscrotal junction, and 30% in the pendulous urethra (3). Their incidence is 15–30 times lower than that of posterior urethral valves (PUV). Clinical manifestations of AUV include dysuria, weak urinary stream, urinary incontinence, and penile swelling due to obstruction. Severe obstruction accompanied by significant bilateral hydronephrosis may lead to end-stage renal disease or even bladder rupture (4).

We conducted a case series review of patients with AUV to analyze their presenting characteristics, diagnosis, management, and prognosis. Combined with a literature review, this study summarizes prognostic results, with a particular focus on renal impairment and functional recovery.

## 2 Methods

### 2.1 Clinical data

A retrospective analysis was performed on eight pediatric patients with AUV treated at Wuhan Children's Hospital, Tongji Medical College, Huazhong University of Science and Technology between 2015 and 2025. The patients, with a median age of 3 years (IQR 1 year 3 months to 6 years 3 months), presented with clinical manifestations including voiding difficulties (poor stream, dribbling), recurrent urinary tract infections, urethral obstruction, and diverticula. The presence of urethral valves or diverticula was confirmed in all cases by retrograde cystography and cystoscopy. Among them, there were four cases of isolated AUV (one concurrent with PUV) and four cases of AUV complicated by urethral diverticula (one also associated with PUV).

### 2.2 Treatment methods

All eight patients underwent surgical intervention. The specific procedures were as follows:

Among the four cases with isolated AUV: Three were treated successfully with cystoscopic electroresection of the valves. One patient, whose valve was located near the urethral meatus, presented with acute urethral obstruction, megalourethra, acute urinary retention, and renal impairment. This case was managed initially with a cystostomy, followed by a second-stage procedure involving valve resection and urethroplasty.
Among the four cases with AUV complicated by anterior urethral diverticula (AUD): Three patients underwent primary valve resection, diverticulectomy, and urethroplasty. One 17-day-old neonate, presenting with a complex condition involving AUV, AUD, and PUV, underwent simultaneous cystoscopic resection of both the anterior and posterior urethral valves along with a cystostomy at 1 month and 17 days of age. The cystostomy tube was dislodged approximately two months postoperatively, necessitating a repeat cystostomy.

### 2.3 Postoperative follow-up

Following the operation, the patient underwent surveillance with VCUG and Renal function test. The median follow-up duration was 2 years and 6 months, with an IQR of 6 months to 4 years.

## 3 Results

**Figure 1:**
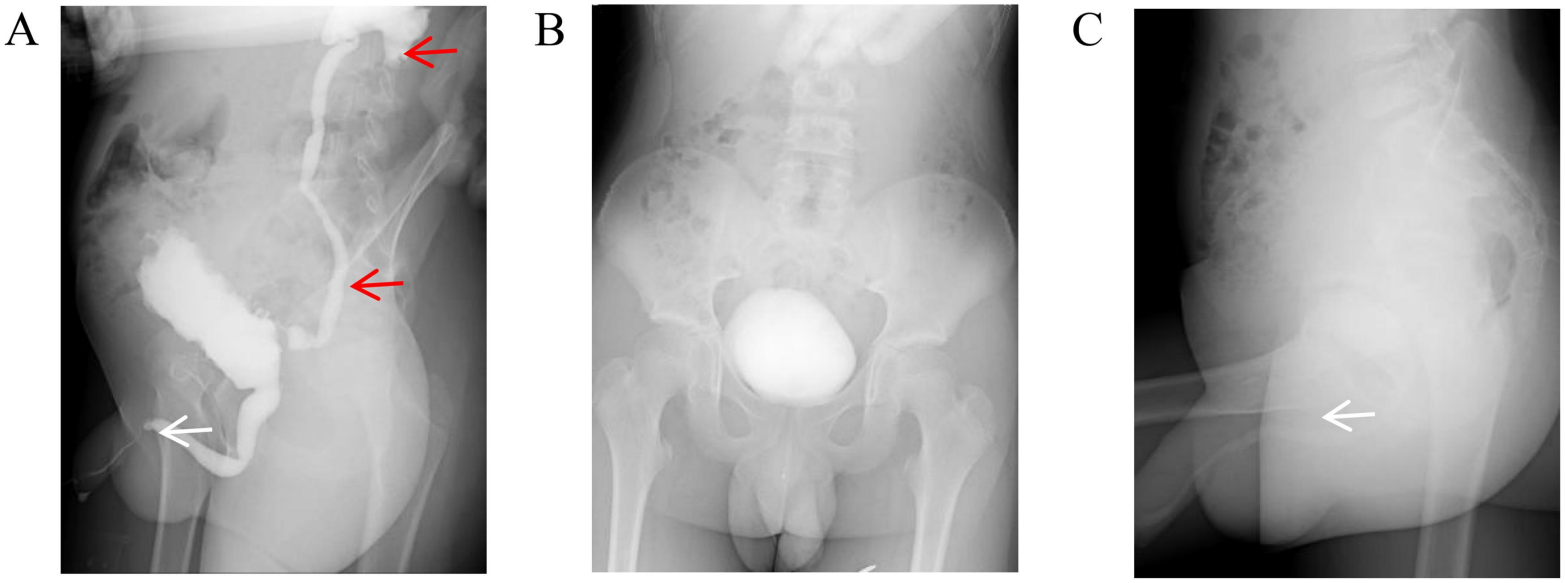
**(A)** VCUG reveals loss of the normal bladder contour with mucosal thickening, bladder trabeculations, and diverticula. Grade III left vesicoureteral reflux is noted (red arrows). The voiding phase demonstrates a distal stricture in the spongy urethra (white arrow), appearing as a thin thread-like segment, accompanied by dilation of the posterior urethra and proximal spongy urethra, as well as impaired bladder emptying. **(B)** Three years postoperatively, the bladder wall appears smooth with no significant signs of VUR. **(C)** The voiding phase shows a patent urethra with normal morphology and no significant obstruction (white arrow), along with normal bladder emptying.

Among the eight patients, the lesion locations were as follows (Table 1): two at the urethral meatus, five at the penoscrotal junction, and one in the bulbar urethra. Retrograde cystography revealed bladder trabeculation in four cases and vesicoureteral reflux (VUR) in another four. Renal impairment was present in three patients. Postoperatively, five of the eight patients recovered completely. In the cases with VUR, the reflux resolved completely following surgical management, as demonstrated in Figures 1A–C.

**Table 1:** Clinical presentation, radiological findings, treatment methods and prognosis in AUD/AUV patients.

Case No	Age	AUV/AUD	Site of AUV	Associated anomalies	Trab	VUR before surgery	Impaired renal function	Surgical intervention	Second surgery	Follow-up duration	prognosis
1	1y7mo	AUV	PJ	H, UTI	+	NV	-	TUF	NV	6y	good
2	10y10mo	AUV	PJ	H, UTI	+	Left (grade III)	-	TUF	NV	5y	good
3	2mo	AUV	PU	H, UTI, AUR, megalourethra, renal failure	+	NV	CR 5.09 mg/dL	V, TUR	NV	1y	UTI
4	3y9mo	AUV	PJ	PUV, UTI, AUR	-	Bilateral (grade III)	Total bilateral ERPF: 119.6 mL/min, Left eGFR 30.26%	TUF	TUD (6y）	4y	Remission, left (grade II), right (grade I), Total bilateral ERPF: 77.23 mL/min; Left eGFR 33.70%
5	5y9mo	AUV/AUD	PU	Distortion penis	-	NV	-	TUD	NV	10y	good
6	2y3mo	AUV/AUD	PJ	H, UTI	-	Left (grade III)	-	TUR + TUD	NV	8y	good
7	7y10mo	AUV/AUD	BU	UTI, spina bifida occulta	-	NV	-	TUR + TUD	NV	7y	good
8	17d	AUV/AUD	PJ	PUD, UTI	+	Left (IV)	Total bilateral ERPF: 77.15 mL/min, Left eGFR ratio: 38.89%	TUF + V	V (2mo)	5mo	Remission, Right (IV)

AUV, anterior urethral valves; AUD, anterior urethral diverticulum; PUV, posterior urethral valve; PJ penoscrotal junction; PU, pendulous urethra; BU, bulbar urethra; AUR, acute urinary retention; H, hydronephrosis; UTI, urinary tract infection; VUR, vesicoureteral reflux; CR, creatinine; eGFR, estimate glomerular filtration rate; TUF, transurethral fulguration of valves; TUR, transurethral resection of valves; V, vesicostomy.

Of the three patients with renal impairment, one presented with acute kidney injury (AKI), defined as a serum creatinine increase of ≥0.3 mg/dL within 48 h. His renal function normalized following the relief of obstruction. The other two patients were diagnosed with chronic kidney disease (CKD), with a glomerular filtration rate (eGFR) is below 60 mL/min/1.73m^2^, or a unilateral GFR proportion 40% of total renal function, with abnormalities present for at least 3 months.

**Figure 2:**
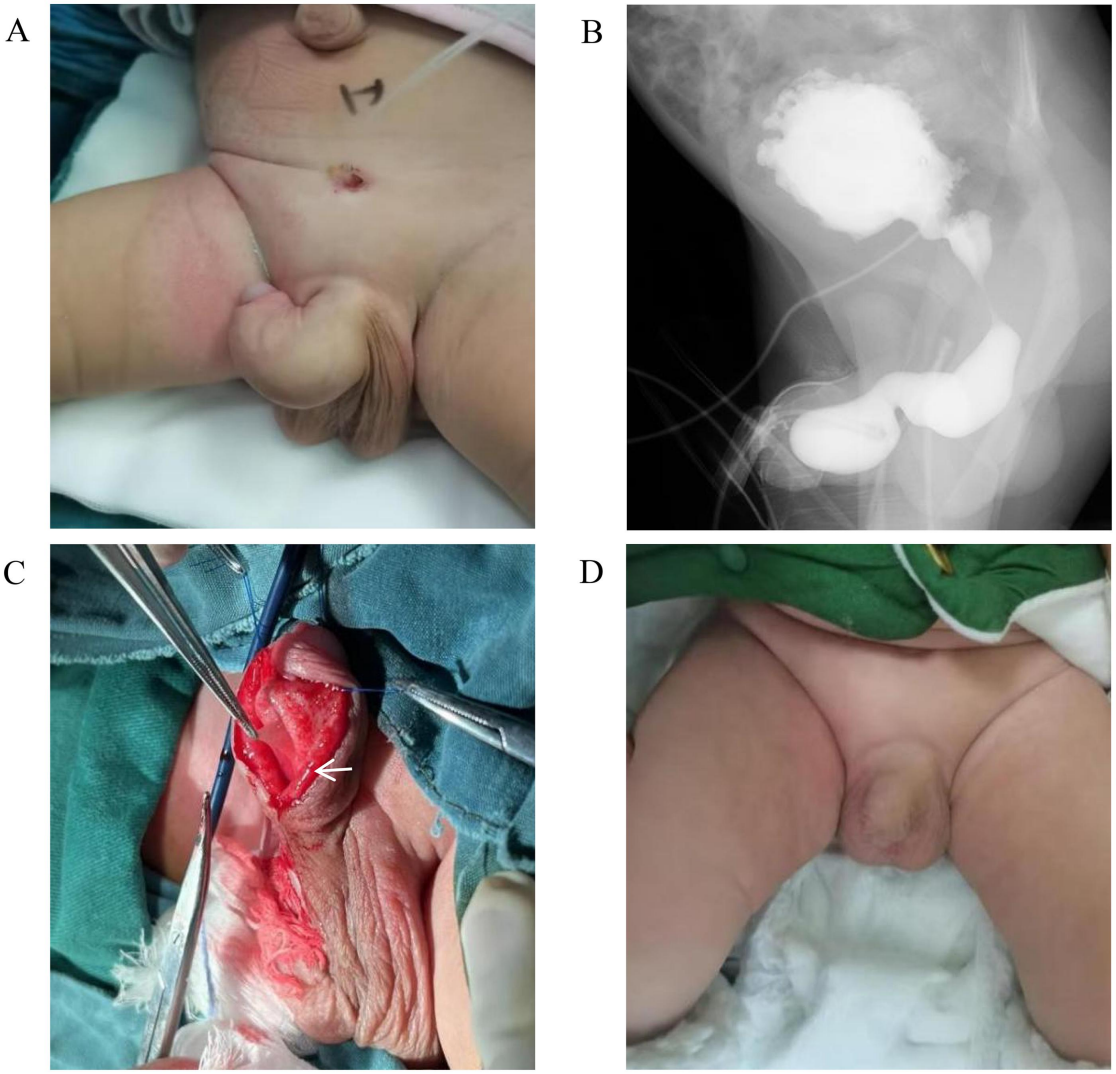
**(A)** An emergency suprapubic bladder puncture and drainage was performed to relieve bladder pressure; however, significant penile swelling persisted. **(B)** VCUG revealed an irregular bladder wall with a trabeculated appearance. Cystic dilation was observed in the anterior urethra, with a maximum internal diameter of approximately 1.1 cm. **(C)** Intraoperative view showing the dilated urethra and a valve-like structure (white arrow). **(D)** Postoperative view showing unobstructed voiding and normal penile appearance.

Case 3, a 2-month-old infant, was prenatally diagnosed with bilateral hydronephrosis at 36 weeks’ gestation, with concomitant oligohydramnios. Attempts to insert a F6 urinary catheter were unsuccessful beyond the penoscrotal junction. Renal function tests showed creatinine (CR) 5.09 mg/dL, urea nitrogen (BUN) 845.60 mg/dL. Emergency suprapubic bladder puncture was performed to decompress the bladder (Figure 2A). Subsequent contrast imaging via the cystostomy tube revealed an irregular bladder wall with multiple diverticula and cystic dilation of the distal spongy urethra, suggestive of anterior urethral valves (Figure 2B). The renal cortex showed poor radiotracer uptake and heterogeneous distribution, with delayed tracer accumulation and clearance in the left kidney. A 7Fr ureteroscope was advanced to the urethral orifice, revealing a valvular stricture obstruction in the anterior urethra, which made insertion of the ureteroscope difficult. A longitudinal skin incision was made along the ventral aspect of the penis at the site of dilation. The subcutaneous fascial tissue was dissected layer by layer until the urethral valve stricture was exposed and valve removal and urethral reconstruction surgery (Figure 2C). One year after the surgery, the child exhibited normal urination, and the penis had returned to its normal appearance (Figure 2D). BUN and CR levels have returned to normal. Postoperatively, homogeneous cortical tracer distribution was noted, with no focal defects. Cystography revealed focal irregularity of the bladder wall with no evidence of vesicoureteral reflux. Occasional urinary tract infections occurred and resolved with antibiotic therapy.

Case 4 presented with bilateral grade III vesicoureteral reflux (VUR). The patient underwent cystoscopic electrocoagulation for both anterior and posterior urethral valves at the age of 3 years and 9 months. A follow-up examination at age 6 showed split renal function on dynamic renal scintigraphy: 34.31% for the left kidney and 65.69% for the right kidney. Additionally, retrograde cystography identified a urethral diverticulum. Subsequent urethral exploration showed postoperative scar tissue hyperplasia at the site of the previous anterior urethral valves. A procedure involving resection of the scar tissue and the diverticulum was performed, followed by urethroplasty. Six months post-surgery, follow-up assessment showed left VUR improved to grade II and right VUR to grade I. Renal function remained stable, with a left GFR of 33.70% and a right GFR of 66.30%. Left renal cortical defects were noted on renal static imaging and the right kidney appeared normal.

**Figure 3:**
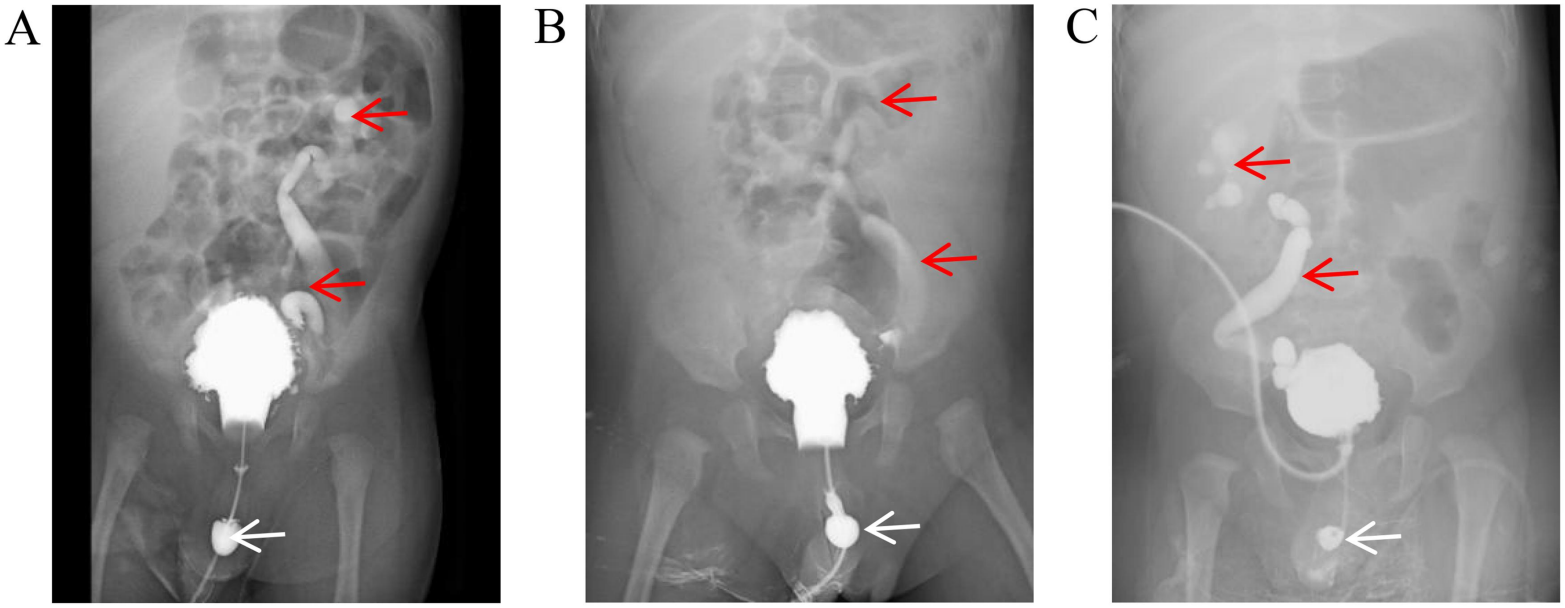
**(A)** VCUG reveals a coarse bladder wall with trabeculation and multiple diverticula of varying sizes at the margins. Grade IV left VUR is present, accompanied by dilation of the left ureter and renal pelvis (red arrows). A prominent diverticulum is observed in the spongy urethra (white arrow). **(B)** Three months postoperatively, the bladder wall remains coarse with persistent diverticula. Dilatation of the left ureter and renal calyces is still evident (red arrows), along with VUR. Localized contrast accumulation is seen in the spongy urethra (white arrow). **(C)** Fistulography performed five months postoperatively shows contrast agent excretion via the urethra. The bladder wall appears irregular, with right-sided VUR (red arrows) graded as level IV. A urethral diverticulum remains visible (white arrow).

Case 8 was a 17-day-old neonate. Initial renal scintigraphy showed a left GFR of 38.89% and a right GFR of 61.11%, accompanied by left-sided grade IV VUR. The case showed impaired left renal cortical function and multiple severe cortical lesions. The primary management consisted of cystoscopic electrocoagulation of the urethral valves and a cystostomy. However, follow-up examinations at 3 and 5 months postoperatively revealed a persistent significant urethral diverticulum and VUR (Figures 3A–C). The cystostomy has been maintained for urinary diversion, and the patient continues to be under close monitoring.

## 4 Discussion

AUVs are a relatively rare congenital genitourinary anomaly in children. The primary clinical manifestations include voiding dysfunction and urinary tract infections. Complete urethral obstruction leading to renal failure is uncommon but underscores the necessity of early diagnosis and intervention.

AUVs are obstructive mucosal folds that cause dilation of the proximal urethra. When isolated, they form a pseudodiverticulum: obstruction leads to urethral distension, and the intact corpus spongiosum prevents full-thickness herniation of the urethral wall. By contrast, congenital deficiency of the corpus spongiosum results in outward bulging of the entire urethral wall, giving rise to a true diverticulum. During endoscopic examination, the diverticulum appears distended with urine, showing a visible opening and bulging beyond the corpus spongiosum (5). McLellan et al. suggested that AUD may result from rupture of dilated bulbourethral glands (6). In the one-month-old infant we treated, the primary clinical manifestations included penile swelling and voiding difficulty, leading to megalourethra. Ultrasound examination showed no abnormalities in the corpus cavernosum. Upon admission, the patient presented with significant renal impairment. Endoscopic evaluation revealed only urethral valves located close to the meatus, consistent with a diagnosis of AUV.

The most critical factor influencing the prognosis of anterior urethral valves is the degree of renal function impairment. Moaddab et al. (7) conducted a retrospective analysis of medical records from 1989 to 2014, summarizing 50 cases of prenatally diagnosed megalourethra. The study found that 41.9% of neonates exhibited renal impairment. Factors such as oligohydramnios, bilateral hydroureter, and earlier gestational age at delivery were associated with neonatal mortality and renal damage. Perlman et al. (8) reported six cases of AUVs detected prenatally. The presence of hydroureter and bladder dilation indicated worsening renal function either *in utero* or in the early postnatal period. Four of these infants developed renal failure during gestation or shortly after birth. Among them, two pregnancies were terminated, one child developed renal failure at 4 years of age, and one infant—whose serum creatinine was 1.1 mg/dL at birth—underwent diverticulectomy and urethral reconstruction. However, follow-up at 14 months showed poor bladder emptying, requiring clean intermittent catheterization. Although serum creatinine remained within the normal range postoperatively, the patient experienced recurrent urinary tract infections, hyperkalemia, and failure to thrive. Sheth et al. (9) reported five cases of prenatally diagnosed AUV, two of which exhibited poor renal outcomes. Among these, two patients developed CKD stage 3a or higher after birth. Notably, one patient who underwent intrauterine vesicoamniotic shunt maintained only stage 1 CKD at the last follow-up, suggesting that early intervention may help preserve renal function and prevent progressive kidney injury.

In our series of 8 patients, 3 cases presented with renal impairment, among which 2 were under 2 months of age. An infant presented with significant oligohydramnios prenatally. Postnatal examination revealed hydroureter and a serum creatinine level of 5.09 mg/dL. Following early bladder diversion, creatinine levels returned to normal, indicating that the renal impairment was likely due to acute obstruction and reversible upon decompression. After secondary valve ablation and urethral reconstruction, follow-up at one year showed notable improvement in ureteral dilation and hydronephrosis. However, the patient experienced recurrent urinary tract infections postoperatively, underscoring the need for ongoing monitoring of renal function. The other patient was 17 days old at onset, presenting with a combination of urethral valves, urethral diverticulum, and posterior urethral valves. Unilateral split renal function was < 40% on renal dynamic imaging and grade IV VUR was detected on the left side. Following surgery, the decreased bladder pressure led to gradual resolution and complete disappearance of the left VUR. However, follow-up examination at 5 months revealed newly developed grade IV VUR on the right side, together with persistent urethral diverticulum, indicating the recurrence of high bladder pressure. Whether his renal function can be reversed remains uncertain and requires close follow-up and observation. The third patient with renal impairment required a second surgery 3 years after the initial procedure due to scar formation at the first operative site. Although the eGFR has not improved immediately after the second surgery, the bilateral VUR has significantly improved.

Irreversible renal damage caused by early obstruction ultimately leads to renal failure, necessitating transplantation or resulting in death. In a meta-analysis of AUV conducted by Routh et al. (2), which reviewed literature published before 2008, 139 cases had completed renal outcome data. Among these, 31 cases (22%) did not achieve restoration of normal renal function. This included 17 patients with stable azotemia, 5 (4%) who required dialysis or transplantation due to end-stage renal disease, and 9 (6%) who died. Pretreatment azotemia, vesicoureteral reflux, and urinary tract infection were identified as factors associated with renal failure and mortality. The simultaneous presence of all three factors increased the odds of poor renal outcomes more than 25-fold (2). Cruz-Diaz et al. (10) reported 11 cases of AUVs, among which early urethral obstruction led to end-stage renal disease in two patients (18%). Menon et al. demonstrated that a preoperative serum creatinine level greater than 1 mg/dL, bladder trabeculation, dilated posterior urethra on VCUG, and bilateral renal impairment were significantly associated with a postoperative eGFR below 60 mL/min/1.73 m^2^. Some studies indicate that compared to PUV, fewer patients with AUV develop CKD stage 3 or progress to end-stage renal disease (ESRD) requiring renal transplantation (7.7% vs. 19.2%), which is consistent with the more favorable long-term outcomes observed in AUV patients. This difference is likely due to the more distal obstruction site in AUV, where an enlarged urethral diverticulum may help dissipate excess pressure upstream of the bladder neck (11, 12). However, some studies have reported a comparable incidence of CKD and ESRD between AUV and PUV patients, suggesting similar rates of progression to advanced renal disease.

VCUG is the primary diagnostic modality for anterior urethral valves. It may demonstrate the following features: dilation or elongation of the posterior urethra, dilatation of the anterior urethra, bladder trabeculation, bladder neck hypertrophy, Vesicoureteral reflux (VUR), Urethral diverticula (3). On VCUG, the urethra appears dilated proximal to the valve and narrowed distal to it. Although cystourethroscopy can visually identify sharp, valve-like obstructive folds in the anterior urethra, these structures may be overlooked during retrograde examination due to effacement of the valves against the urethral wall.

Treatment of AUV varies depending on the patient's age, the severity of upper urinary tract damage, and the extent of anterior urethral malformation. In most cases, endoscopic electrocautery or resection using a hooked electrode can be performed to disrupt the valves. In severe cases accompanied by a urethral diverticulum, a small diverticulum may be managed by incising its distal lip to relieve obstruction. However, Sheth et al. recommend that in the presence of a large diverticulum, open surgical diverticulectomy with urethral reconstruction should be performed directly, rather than attempting endoscopic intervention (9). Some experts also recommend initial endoscopic valve ablation, with diverticulectomy reserved for cases where endoscopic management fails (5). Prenatally diagnosed anterior urethral valves are typically associated with bilateral hydronephrosis. In severe cases, megaureter and/or megacystis may also develop (13). Fetal lower urinary tract obstruction may be managed with intrauterine vesicoamniotic shunt placement, which has been shown to improve perinatal survival (14). For preterm infants or neonates, vesicostomy may be required to relieve obstruction until the infant is mature enough to undergo definitive endoscopic or reconstructive procedures.

## 5 Conclusion

AUVs are a rare form of urethral obstruction. Early diagnosis and treatment are associated with a favorable prognosis. However, if complicated by renal impairment, the condition may lead to renal failure requiring dialysis or even renal transplantation, and can potentially result in death, indicating a poor prognosis.

## Data Availability

The original contributions presented in the study are included in the article/Supplementary Material, further inquiries can be directed to the corresponding authors.

## References

[1] Paulhac P Fourcade L Lesaux N Alain JL Colombeau P. Anterior urethral valves and diverticula. BJU Int. (2003) 92(5):506–9. 10.1046/j.1464-410X.2003.04380.x 12930408

[2] Routh JC Mcgee SM Ashley RA Reinberg Y Vandersteen DR. Predicting renal outcomes in children with anterior urethral valves: a systematic review. J Urol. (2010) 184(4 Suppl):1615–9. 10.1016/j.juro.2010.03.119 20728183

[3] Parmar JP Mohan C Vora M. Anterior urethral valve: a rare but an important cause of infravesical urinary tract obstruction. Pol J Radiol. (2016) 81:209–11. 10.12659/PJR.896230 27231492 PMC4865270

[4] Punekar SV Rao NR Kelkar AR Gavande PM Prem AR. Anterior urethral valve in an adolescent boy. J Postgrad Med. (1995) 41(2):46–7. 10707710

[5] Jain P Prasad A Jain S. Are anterior urethral valve and anterior urethral diverticulum two separate entities: a radiological and endoscopic review. J Pediatr Urol. (2021) 17(1):101.e1–e9. 10.1016/j.jpurol.2020.11.002 33229229

[6] Mclellan DL Gaston MV Diamond DA Lebowitz RL Mandell J Atala A. Anterior urethral valves and diverticula in children: a result of ruptured Cowper’s duct cyst? BJU Int. (2004) 94(3):375–8. 10.1111/j.1464-410X.2004.04854.x 15291870

[7] Moaddab A Sananes N Hernandez-Ruano S Werneck Britto IS Blumenfeld Y Stoll F. Prenatal diagnosis and perinatal outcomes of congenital megalourethra: a multicenter cohort study and systematic review of the literature. J Ultrasound Med. (2015) 34(11):2057–64. 10.7863/ultra.14.12064 26446816

[8] Perlman S Borovitz Y Ben-Meir D Hazan Y Nagar R Bardin R. Prenatal diagnosis and postnatal outcome of anterior urethral anomalies. Prenat Diagn. (2020) 40(2):191–6. 10.1002/pd.5582 31654578

[9] Sheth KR White JT Bilgutay AN Seth A Mittal AG. Anterior urethral valves - a rare but challenging congenital pathology. J Pediatr Urol. (2020) 16(5):585.e1–e7. 10.1016/j.jpurol.2020.03.024 32340880

[10] Cruz-Diaz O Salomon A Rosenberg E Moldes JM De Badiola F Labbie AS. Anterior urethral valves: not such a benign condition…. Front Pediatr. (2013) 1:35. 10.3389/fped.2013.00035 24400281 PMC3864262

[11] Kajbafzadeh AM Payabvash S Karimian G. Urodynamic changes in patients with anterior urethral valves: before and after endoscopic valve ablation. J Pediatr Urol. (2007) 3(4):295–300. 10.1016/j.jpurol.2006.11.002 18947759

[12] Kajbafzadeh AM Hosseini Sharifi SH Keihani S Soltani MH Tajali A Salavati A. Concomitant anterior and posterior urethral valves in pediatrics: a single center experience over 12 years and long-term follow up after endoscopic treatment. Int J Urol. (2015) 22(5):514–9. 10.1111/iju.12712 25689730

[13] Salem AB Mazhoud I Laamiri R Salem R Laajili H Sahnoun L. Anterior urethral valve: uncommon association with renal duplicity. J Neonatal Surg. (2017) 6(2):41. 10.21699/jns.v6i2.544 28770138 PMC5538606

[14] Kohaut J Holtkamp G Fischer-Mertens J Schulten D Kohl S Habbig S. A new spectrum of neonatal urethral pathologies in the era of early vesicoamniotic shunting? World J Urol. (2024) 42(1):589. 10.1007/s00345-024-05307-4 39441227

